# Lymphoepithelioma-like carcinoma in liver not associated with Epstein-Barr virus: a report of 3 cases and literature review

**DOI:** 10.1186/s13000-020-01035-6

**Published:** 2020-09-23

**Authors:** Kai Zhang, Changcheng Tao, Zonggui Tao, Fan Wu, Songlin An, Jianxiong Wu, Weiqi Rong

**Affiliations:** 1 Department of Hepatobiliary Surgery, National Cancer Center/National Clinical Research Center for Cancer/Cancer Hospital, Chinese Academy of Medical Sciences and Peking Union Medical College, Beijing, 100021 China; 2 Department of Imaging, Jinan city people’s hospital, Shandong First Medical University, Jinan, 271199 China; 3 Department of Peritoneal Cancer Surgery, Beijing Shijitan Hospital, Capital Medical University, Beijing, 100038 China

**Keywords:** Liver Cancer, Lymphoepithelioma-like carcinoma, Hepatocellular carcinoma, Case report

## Abstract

**Background:**

Lymphoepithelioma-like carcinoma is a rare distinctive variant of liver cancer with unique epidemiological and pathological characteristics, characterized by dense lymphocyte infiltration. It can be divided into lymphoepithelioma-like hepatocellular carcinoma and lymphoepithelioma-like intrahepatic cholangiocarcinoma. Existing research shows that the prognosis of this tumor is good. To date, only 101 cases have been reported.

**Case presentation:**

The first patient was a 62-year-old Chinese man with hepatitis B virus infection who presented with a single lesion in the liver. The patient underwent surgical treatment and was discharged on the 4th day. The patient was diagnosed with combined lymphoepithelioma-like hepatocellular carcinoma and cholangiocarcinoma; he has been alive for 15 months. The second patient was a 63-year-old Chinese woman with right upper abdominal pain and hepatitis B virus infection. The imaging examination revealed a single lesion in the liver. The patient underwent surgical treatment and was discharged 1 week later. The patient was diagnosed with lymphoepithelioma-like hepatocellular carcinoma and was considered to have recurrence in the lymph nodes approximately 2 years after the operation. The patient underwent local radiotherapy; she has been alive for 60 months. The third patient was a 50-year-old Chinese man with hepatitis B virus infection who presented with a single lesion in the liver and two enlarged lymph nodes. The patient received liver puncture before surgery to indicate lymph node metastasis and experienced local recurrence after liver resection. The patient underwent chemotherapy and radiotherapy. The patient was diagnosed with lymphoepithelioma-like hepatocellular carcinoma. The patient was deceased at 24-month follow-up.

**Conclusions:**

This article reports 3 cases without Epstein-Barr virus and reviews the current literature, which suggests even mixed pathological type or locally advanced cases of LELC with lymph node metastasis and postoperative recurrence should be actively treated for a longer survival period.

## Background

At present, liver cancer is now the second leading cause of cancer-related death in the world [1]. Primary liver cancer is one of the most common malignant tumors of the digestive system in China, being third in incidence and fourth in mortality [2]. Hepatocellular carcinoma (HCC) and intrahepatic cholangiocarcinoma (ICC) represent two major types of primary liver cancer. Because most patients are diagnosed at an advanced stage, the treatment options available are very limited. In the United States, a 5-year survival rate of 16% for liver cancer has been reported, and it has become the most aggressive malignancy after pancreatic cancer [3].

**Fig. 1:**
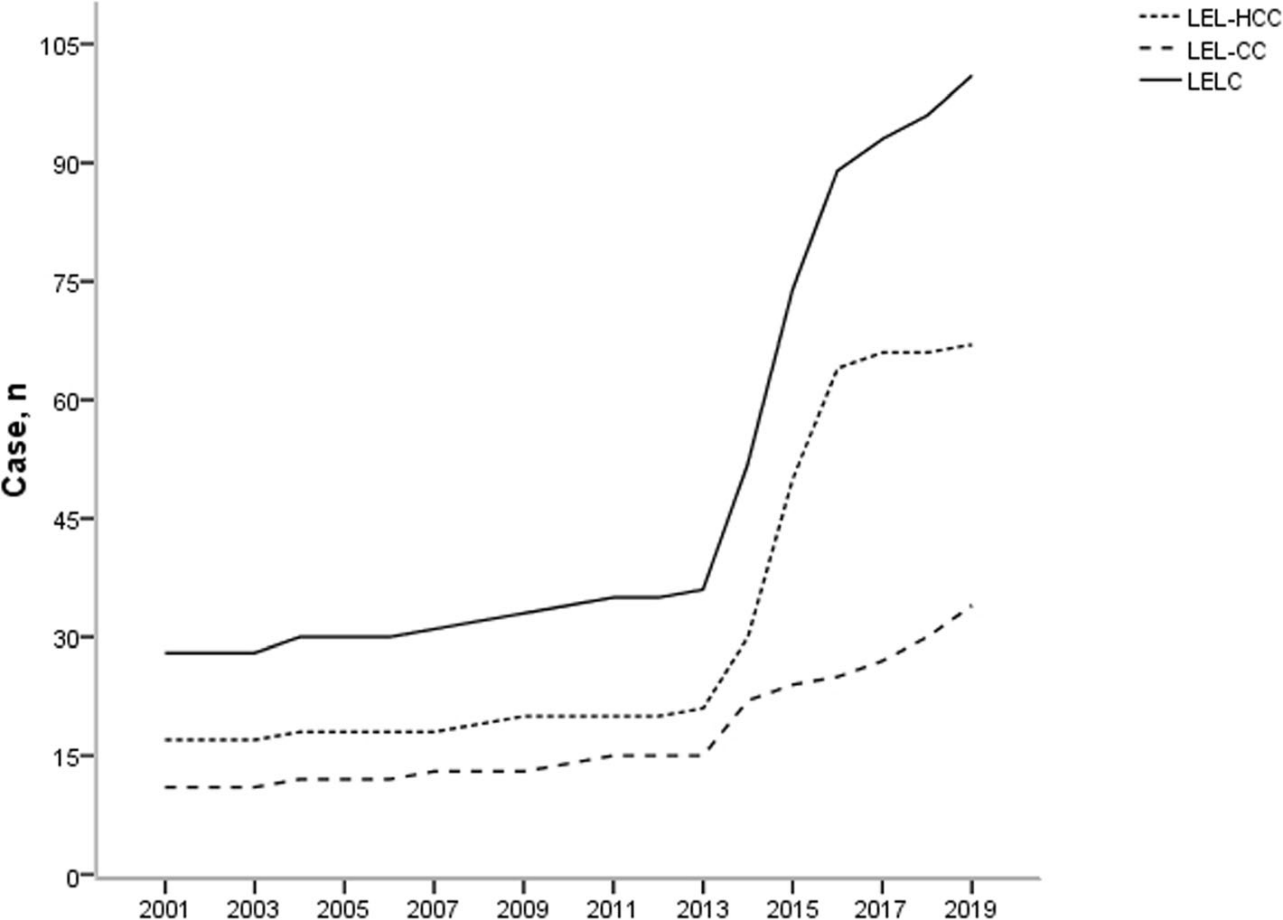
Number of reported cases of lymphoepithelioma-like carcinoma (LELC). Number of cases reported in the English literature since 2001 for lymphoepithelioma-like hepatocellular carcinoma (LEL-HCC), lymphoepithelioma-like cholangiocarcinoma (LEL-CC), and both types

Lymphoepithelioma-like carcinoma (LELC) is a type of tumor composed of undifferentiated epithelial cells, with obvious lymphoid infiltration, which was originally used to describe a tumor in the nasopharynx. Such tumors in other organs, including the lung, breast, prostate, bladder, uterus and liver, have since been reported [4–9]. Similar to primary liver cancer, LELC can be classified as lymphoepithelioma-like hepatocellular carcinoma (LEL-HCC) and lymphoepithelioma-like intrahepatic cholangiocarcinoma (LEL-CC) [10]. In 1998, Wada et al [11] defined LELC as the presence of more than 100 tumor infiltrating lymphocytes in 10 high power fields, but no unified definition has been established. Only a few cases have been reported since this period. LELC was acknowledged by the World Health Organization (WHO) as a distinctive variant of liver cancer in 2010 [12]. Although lymphocyte infiltration needs to be observed for its diagnosis, but the density of lymphocyte infiltration required for further diagnosis is not clear. Therefore, the definition and pathological classification of LELC are still under study. LELC is a relatively rare finding, though there has been a significant increase in the number of reported cases over the past few years (Fig. 1). From 1998 to 2020, 41 studies have been published and 67 cases of LEL-HCC and 34 cases of LEL-CC have been reported.

At present, it is believed that LELC has unique epidemiological and pathological characteristics. Compared with typical HCC and ICC, the prognosis of LELC is good. LELC may be related to a large amount of lymphocyte infiltration [10], and the lymphocytic infiltration is this distinctive variant of liver cancer may be related to immune response. Overall, its pathogenesis and factors affecting prognosis deserve further study.

This article reports 3 cases of LELC and reviews the current literature on LELC in terms of epidemiology, clinical treatment, pathology and research prospects.

## Case presentation

### First case

**Fig. 2:**
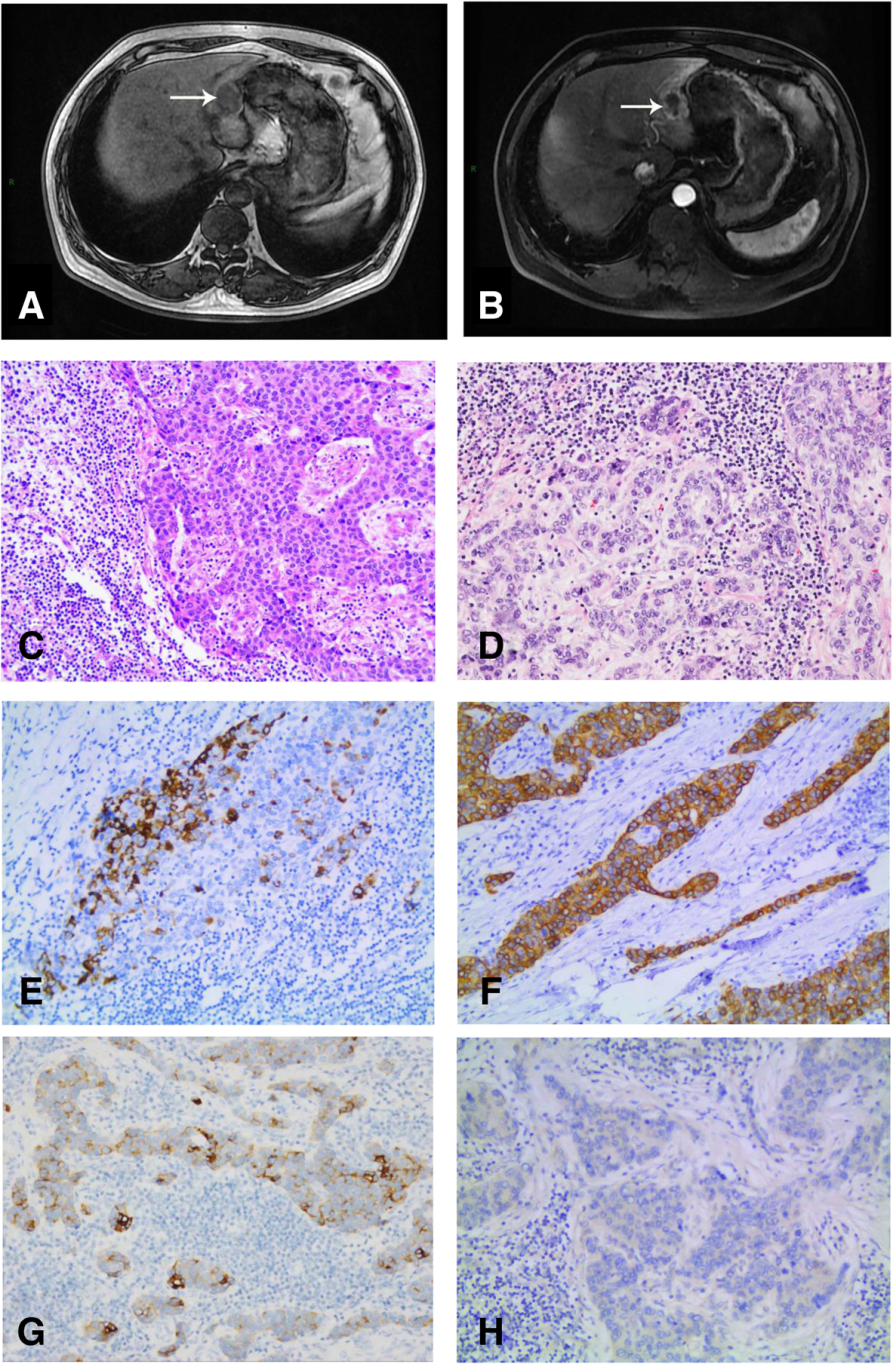
Preoperative MRI findings and microscopic findings of the resected specimen. MRI showing a space-occupying lesion of approximately 3.2 × 2.5 cm in left lateral lobe, hypointense on T1-weighted images (**a**), enhancement in arterial phase (**b**). Tumor is composed of poorly differentiated cells admixed with significant lymphocytic infifiltrates (**c**, HE, × 200). Histological findings showing glandular differentiation (**d**, HE, × 200). Immunohistochemical staining for hepatocyte, CK18, CK19 is positive (**e**, HE, × 200), (**f**, HE, × 200), (**g**, HE, × 200). Immunohistochemical staining for EBER is negative (**h**, HE, × 200)

The first patient was a 62-year-old Chinese man who was hospitalized on September 5, 2018, due to the a high AFP level was found during a routine physical examination and a liver tumor indicated by abdominal ultrasound. Laboratory examination showed the following: AFP 19.5 ng/ml (0–7 ng/ml); CEA 5.31 ng/ml (0–5 ng/ml); CA199 17.26 U/ml (0–37 U/ml). Hepatitis B examination showed that HBsAb(+), HBeAb(+), and HBcAb(+). There were no abnormality in routine blood test. Blood biochemical tests showed DBIL of 5.9 μmol/L (0–5.1 μmol/L) and IBIL of 13.70 μmol/L (0–11.97 μmol/L). A coagulation test showed a plasma D-dimer level of 0.61 mg/L FEU (0–0.55 mg/L FEU). Abdominal MRI suggested a liver left lateral lobe space-occupying lesion, about 3.2 × 2.5 cm (Fig. 2a, b). According to PET-CT, there was a slightly low-density nodular shadow under the capsule of the left outer lobe of the liver, of approximately 2.8 × 2.4 cm, and the boundary was unclear. Radiation uptake was increased, and the maximum SUV was 4.0. It was regarded as a malignant lesions, and the radiation uptake of the remaining liver parenchyma was no clearly different. Multiple lymph nodes were found in the abdomen, with some increase in radioactivity uptake. The maximum SUV is 4.6; the large lymph node diameter was approximately 0.9 cm. No other extrahepatic tumor was found by imaging.

After discussion by a multidisciplinary team (MDT), we decided to perform surgery for the patient using laparoscopic left lateral lobectomy of the liver. Intraoperative findings were as follows: no ascites; no nodules in the abdominal and pelvic peritoneum; no abnormalities in the spleen, stomach, small intestine or large intestine; and no obvious expansion of the gallbladder or common bile duct. The appearance of the gallbladder was normal, and no stones or masses were found. Enlarged lymph nodes were not detected around the hepatoduodenal ligament. The liver was soft without obvious cirrhosis. The tumor was located in the left lateral lobe. It was tough and protruded from the surface of the liver. The rest of the liver was normal. Macroscopically, a 16 × 8 × 4 cm segment of the liver was resected, and a 2.8 × 2.5 × 2.5 cm gray-white hard tumor mass was present in the section. The pathologic diagnosis was lymphoepithelioma-like carcinoma (LELC); it was mainly hepatocellular carcinoma with differentiation of scattered cholangiocarcinoma, largely solid nest-like (Fig. 2c, d). The Edmondson-Steiner stage was III (poorly differentiated). The tumor was a small cancer involving the liver capsule without clear necrosis or microvascular invasion (MVI:0). There was no satellite nodule in the surrounding liver tissue and no cancer at the cutting edge of the liver. The TNM stage was T1N0M0, and the BCLC stage was A. Immunochemistry analysis showed AFP(−), Arg-1(−), CA199(−), CK18(3+), CK19(1+), CK7(1+), GPC3(3+), hepatocyte(+) and EBER ISH(−) (Fig. 2e, f, g, h). This case can be diagnosed as LELC. The operation was successful, and the patient was discharged on the 4th day after the operation. After MDT discussion, we decided to perform regular observation rather than postoperative adjuvant treatment according to the pathological diagnosis. The patient has been alive for 15 months since the surgery, and the patient’s quality of life is good.

### Second case

**Fig. 3:**
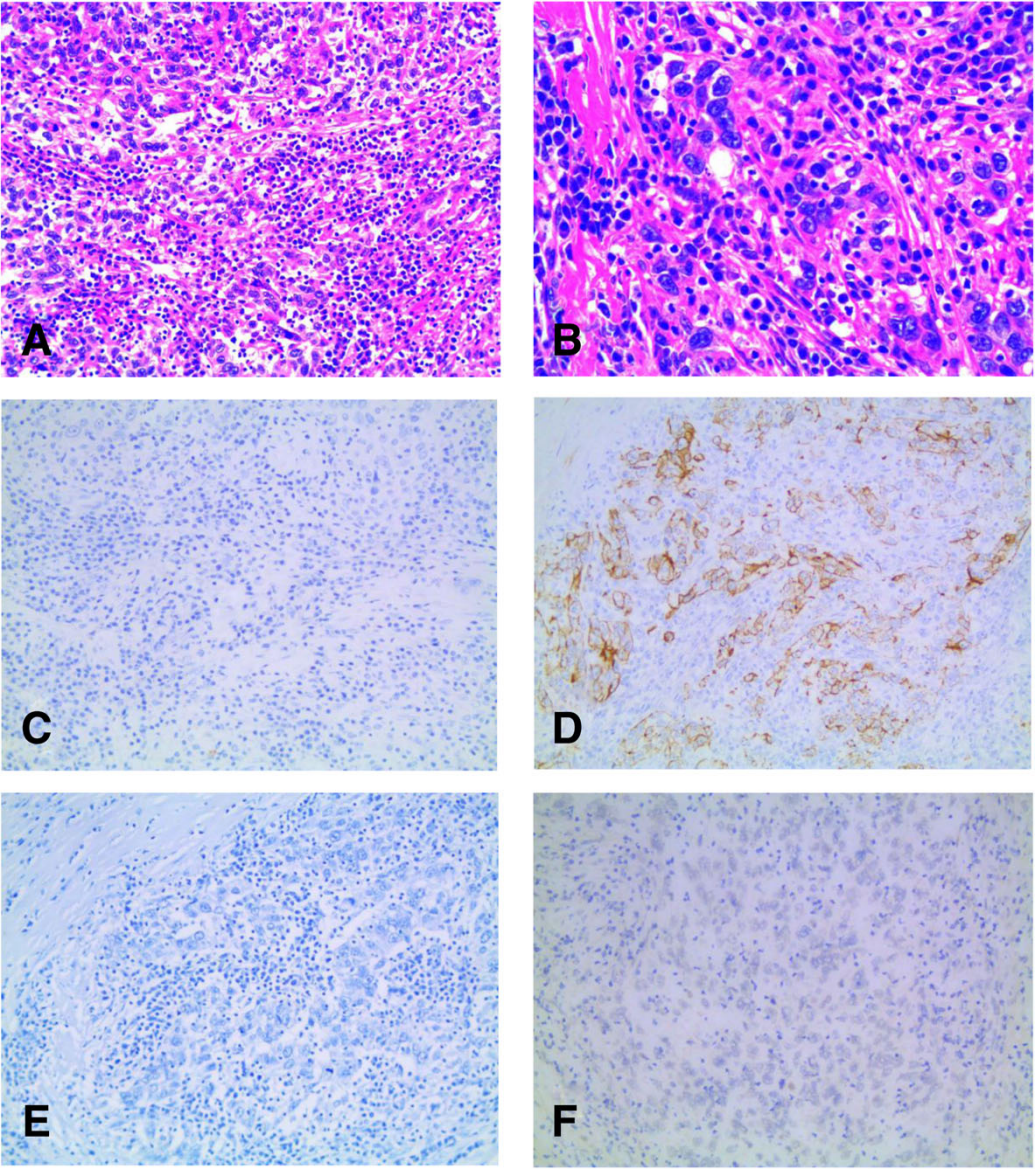
Microscopic findings of the resected specimen. The tumor composed of poorly differentiated epithelial cells with vesicular nuclei, prominent nucleoli and intense lymphocytic infiltration. (**a**, HE, × 200), (**b**, HE, × 400). Immunohistochemical staining for hepatocyte is negative (**c**, HE, × 200). Immunohistochemical staining for CK18 is positive (**d**, HE, × 200). Immunohistochemical staining for Arg and EBER is negative (**e**, HE, × 200), (**f**, HE, × 200)

The second patient was a 63-year-old Chinese woman who was hospitalized on January 22, 2015 due to paroxysmal right upper abdominal pain for 1 year. Hepatitis B examination showed HBsAb(+), HBeAb(+), and HBcAb(+). Laboratory examination showed the following: AFP, 1.49 ng/mL (0–7 ng/mL); CEA, 0.545 ng/mL (0–5 ng/mL); and CA199, 9.02 U/mL (0–37 U/mL). The patient was treated with aspirin after coronary stent implantation for coronary heart disease 3 years prior. The patient’s previous history of hypertension was under good control. The patient underwent hysterectomy 15 years previously for endometriosis. Abdominal MRI suggested an S7 space-occupying lesion, considering the possibility of a malignant tumor, cholangiocarcinoma or metastatic cancer, with liver lymph node metastasis and a slightly larger spleen. Due to the imaging examination of patients was from other hospitals, it is not provided here. On January 27, 2015, the patient underwent irregular resection of the right liver plus hilar lymphadenectomy. Macroscopically, a 7 × 7 × 3.5 cm segment of the liver was resected; a 3.2 × 2 × 2.2 cm gray-white hard tumor mass was found in the section. The tumor boundary was not clear, involving the hepatic capsule, and the closest distance from the base margin was 1.1 cm. There was no obvious nodular change in the surrounding liver. The pathological diagnosis was LELC, mainly hepatocellular carcinoma (Fig. 3a, b). The tumor involved the hepatic capsule, and no cancer was found at the cutting edge of the liver base. Lymphocytes had infiltrated the peripheral hepatic portal area, and no metastatic cancer was found in the lymph nodes removed. The Edmondson-Steiner stage was III (poorly differentiated). The TNM stage was T1N0M0. The BCLC stage was A. Immunochemistry analysis showed CK18(2+), CK7(−), CK19(−), Arg-1(−), CD10(−), CEA(−), hepatocyte(−), AFP(−), EBER(−), CD20(−), CD4(1+) and CD8(−) (Fig. 3c, d, e, f). This case can be diagnosed as LELC.

The patient recovered well and was discharged 1 week later. There was no obvious abnormality upon regular re-examination for more than 1 year. The patient received whole-body PET-CT f on December 26, 2016, which revealed high uptake by multiple lymph nodes in the portal space. The largest SUV was 11.0. The short diameter was approximately 2.5 cm, which was considered to be lymph node recurrence after the operation for liver cancer. After discussion by the MDT, it was considered that surgery would be difficult because of the close proximity of the tumor to blood vessels and the duodenum. It was suggested that local radiotherapy should be performed, and IMRT was performed in our hospital from February 6, 2017, to March 10, 2017. The prescription dose of the first course was 95% pGTV 6.72 Gy/3.36 Gy/2f, 95% PTV 3.6 Gy/1.8 Gy/2f; the prescription dose of the second course was pGTV 55.2 Gy/2.4 Gy/23f, 95% PTV 41.4 Gy/1.8 Gy/23f; 25 doses were completed. Simultaneous Xeloda chemotherapy taken orally twice was used on the day of radiotherapy at a dose of 1650 mg/m2/d. The patient carried out auxiliary acid suppression, mucosal protection, liver protection treatment and was discharged without incident after treatment. After 60 months of follow-up, the patient was in good condition. Disease-free survival (DFS) was 23 months.

### Third case

**Fig. 4:**
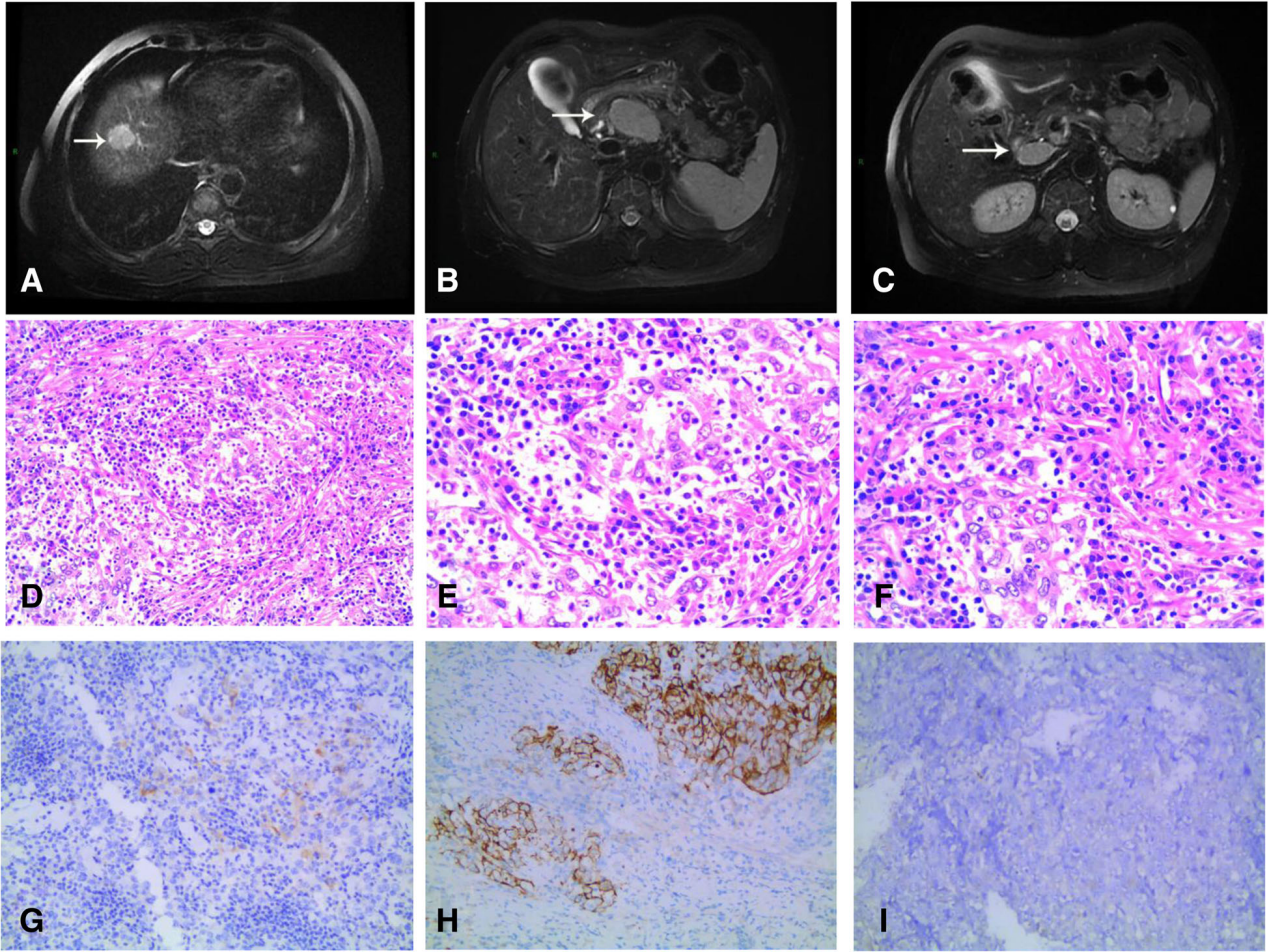
Preoperative MRI findings and microscopic findings of the resected specimen. MRI showing a tumor of approximately 2.7 × 2.2 cm in segment VIII, hypointense on T1-weighted images (**a**). An enlarged lymph node can be seen on the upper margin of the pancreas, beside the common hepatic artery, approximately 5.2 × 3.4 cm in size (**b**). An enlarged lymph node is present beside the portal vein, behind the duodenal ligament (**c**). Proliferation of atypical large cells, with large nuclei and prominent nucleoli, and characterized by an eosinophilic cytoplasm. Epithelial cells were surrounded by a dense lymphoid stroma extending inside the tumor (**d**, HE, × 200), (**e**, HE, × 400), (**f**, HE, × 400). Immunohistochemical staining for AFP and CK19 is positive (**g**, HE, × 200), (**h**, HE, × 200). Immunohistochemical staining for Arg and EBER is negative (**i**, HE, × 200)

The third patient was a 50-year-old Chinese man who was hospitalized on April 29, 2014, due to a liver tumor found during a routine physical examination. We reported this case in 2015 [9]; here, we briefly introduce the course of the disease, focusing on its postoperative treatment and follow-up. Laboratory examination showed HBsAg(+), HBeAb(+), HBcAb(+), HCV-Ab(−), HBV-DNA(−), AFP 31.93 ng/ml (0–7 ng/ml), and CA199 10.51 U/ml (0–37 U/ml). The patient had type II diabetes mellitus, and his blood sugar was well controlled at presentation. Abdominal MRI showed a 2.7 × 2.2 cm right anterior segment tumor with cirrhosis. (Fig. 4a). Multiple enlarged lymph nodes were found in the retroperitoneum, with a maximum of approximately 5.2 × 3.4 cm (Fig. 4b). An enlarged lymph node was also observed behind the duodenal ligament and beside the portal vein (Fig. 4c). Abdominal ultrasound showed a hypoechoic nodule at the liver dome, of approximately 1.9 × 2 cm. A hypoechoic lymph node, of approximately 4.2 × 2.9 cm, was present 5 cm behind the peritoneum, without an obvious blood flow signal. After MDT discussion, percutaneous ultrasound-guided biopsy was recommended for the lesion and lymph node. Based on the pathology results, no cancer cells were found in the liver tumor but were present in the lymph node. PET-CT was also performed, which indicated increasing radioactive uptake, which was considered to be primary liver cancer. The patient underwent hepatectomy of the VIII segment and two enlarged lymph node resections on May 23, 2014. Microscopically, the tumor consisted of poorly differentiated hepatocellular carcinoma with lymphoid infiltration (Fig. 4d, e, f). The Edmondson-Steiner stage was III (poorly differentiated). The lymph nodes removed were infiltrated by hepatocellular cancer cells. Immunochemistry results showed AFP (1+), AE1/AE3 (2+), CK18 (2+), Arg-1(−), hepatocyte(−), CK19 (2+), CK20 (1+), CK7 (−), and EBER (−) (Fig. 4g, h, i). The pathological diagnosis was LELC. The TNM stage was T1N1M0, with BCLC stage C.

After the operation, abdominal CT showed enlarged retroperitoneal lymph nodes, which was considered recurrence. From August 4, 2014, to September 2, 2014, the patient received 3 cycles of oxaliplatin and tegio chemotherapy; from October 5, 2014, the patient received 1 cycle of paclitaxel/cisplatin chemotherapy; from October 21, 2014, to November 14, 2014, the patient received 2 cycles of paclitaxel chemotherapy; from November 28, 2014, to December 28, 2014, the patient received 3 cycles of cisplatin chemotherapy. CT re-examination after nine cycles of chemotherapy indicated that the multiple enlarged retroperitoneal lymph nodes had changed little compared with the anterior lymph nodes. Later, patient was treated with radiotherapy. The following was found for the target area: GTV - retroperitoneal lymph nodes were seen on imaging; PGTV - GTV expanded outward by 0.5 cm; CTV - GTV, hilar and retroperitoneal lymphatic drainage area; PTV - CTV plus 0.5 cm (front, back, left and right) plus 1 cm (up and down). The patient was treated with a dose of 95% of PGTV, 60.2 Gy/2.15 Gy/28F, and 95% of PTV. The radiotherapy was completed on March 5, 2015, and the patient was discharged.

However, the patient was deceased at the 24-month follow-up. The DFS for this case was 1 month, with overall survival (OS) of 24 months. This patient was previously reported by us, and we updated and integrated the information. The detailed clinical data and pathological features of the above three patients are shown in Table 1 and Table 2.

**Table 1:** Demographic and oncological characteristics of the 3 patients

Case	Sex	Age, years	Region	HBV	HCV	Cirrhosis	AFP, > 7 ng/mL	BCLC stage	No. Of lesions	Size,mm	Treatment	Recurrence	FU, mo.	Outcome	DFS, mo.
First	M	62	Asian	+	–	–	+	A	1	28	LR	N	15	AWOD	/
Second	F	63	Asian	+	–	–	–	A	1	32	LR + RT	Y	60	AWOD	23
Third	M	50	Asian	+	–	+	+	C	1	35	LR + CT + RT	Y	24	DOD	1

*F* female, *M* male, + positive, − negative, *AFP* a-fetoprotein, *HBV* hepatitis B virus, *HCV* hepatitis C virus, *N* NO, *Y* Yes, *LR* liver resection, *CT* chemotherapy, *RT* radiotherapy, *AWOD* alive without disease, *DOD* died of the disease, *FU* follow-up, *mo*. months, *DFS* disease-free survival

**Table 2:** Pathological classification and immunohistochemical markers of the 3 patients

Case	Diagnosis	Classification	Lymph node	TNM stage	Edmondson-Steiner stage	AFP	Hepatocyte	Arg	CK7	CK19	CK18	EBER ISH
First	LELC	Hepatocellular&Cholangio-carcinoma differentiation	N	T1N0M0	III (poorly differentiated)	–	+	–	+	+	+	–
Second	LELC	Hepatocellular	N	T1N0M0	III (poorly differentiated)	–	–	–	–	–	+	–
Third	LELC	Hepatocellular	P	T1N1M0	III (poorly differentiated)	1+	–	–	–	+	+	–

*LELC* lymphoepithelioma-like carcinoma, − negative, + positive, *AFP* a-fetoprotein, *EBER* EBV-encoded RNA, *ISH* in situ hybridization, *arg* arginase

All of the above three patients were satisfied with the surgical treatment and no adverse reactions occurred.

## Literature review

LELC is a distinctive variant of liver cancer and can be divided into LEL-HCC and LEL-CC. LELC is a relatively rare finding. From 1998 to 2020, 41 studies have been published, with 67 cases of LEL-HCC and 34 cases of LEL-CC being reported.

### Lymphoepithelioma-like hepatocellular carcinoma (LEL-HCC)

LEL-HCC is one of the pathological classifications of LELC. To date, 67 cases of LEL-HCC have been reported, as detailed in Table 3. The available demographics of the patients and features of the tumors are displayed in Table 4. Calderaro et al [25] reported 13 cases of LEL-HCC treated with liver resection for hepatocellular carcinoma, but no relevant information was provided. According to the current literature analysis, the majority of patients with LEL-HCC are male (63%) and White, with a median age of 58 years old (37–81). Twenty-two cases were complicated with HBV infection, accounting for 41%; 19 with HCV infection, accounting for 35%; and 24 with cirrhosis, accounting for 45%. In total, 21 patients exhibited increased AFP, higher than 20 μg/l, accounting for 51%. Most cases were BCLC stage 0/A; 25% of patients showed vascular invasion but only one patient EBV infection. Most of the patients received surgical treatment, accounting for 91%, and 6 patients received liver transplantation.

**Table 3:** Reported cases of LEL-HCC

Reference	Age/Sex	Race	HBV	HCV	EBV	Liver cirrhosis	AFP, > 20 ng/mL	Tumor location	BCLC (0/A)	Tumor no.	Tumor size(mm)	Vascular invasion	Treatment	Recurrence	FU, months	Outcomes
An et al [9]	50/M	Asian	+	–	–	+	+	R	–	1	35	–	LR	Y	6	Alive
Wang et al [13]	37/F	NA	+	+	–	+	+	R	+	1	32	NA	LR	N	52	Alive
Chen et al [14]	56/M	Asian	–	+	–	+	+	R	+	1	32	–	LR	Y	21	Died
Cacciato Insilla et al [15]	81/F	NA	–	+	–	–	+	L	+	1	72	NA	LR	N	15	Alive
Shinoda et al [16]	79/M	Asian	–	–	–	–	+	L	+	1	42	–	LR	Y	20	Alive
Wei et al [17]	42/F	Asian	–	–	–		+	L	+	1	46	NA	LR	N	8	Alive
Park et al [18]	57/M	Asian	+	–	–	+	–	R	+	1	27	NA	LR	N	60	Alive
Emil et al [19]	50/M	White	+	+	–	+	–	NA	+	1	40	–	OLT	N	120	Alive
	54/M	White	–	–	–	–	–	NA	+	2	20	–	OLT	Y	92	Died
	59/M	White	+	+	–	+	–	NA	–	4	50	–	OLT	N	96	Alive
	45/M	White	–	+	–	+	–	NA	+	1	20	–	OLT	N	56	Alive
	64/M	White	–	–	–	+	–	NA	–	2	40	+	OLT	N	36	Alive
Si et al [20]	39/F	Latino	–	+	+	+	NA	NA	+	1	10	+	OLT	Y	5	Died
Nemolato et al [21]	47/F	NA	–	–	–	–	NA	R	+	1	22	NA	LR	N	15	Alive
Shirabe et al [22]	58/F	Asian	+	–	NA	+	+	R	+	1	22	NA	LR	N	78	Alive
Patel et al [23]	74/F	White	–	–	–	–	–	NA	–	Multi	65	+	LR	Y	24	Died
	65/M	White	–	–	–	–	–	NA	+	1	48	–	LR	N	60	Died
	65/F	White	–	–	–	–	+	NA	+	1	13	–	LR	N	108	Alive
	70/F	White	–	–	–	–	+	NA	+	1	27	–	LR	N	72	Alive
	61/F	White	–	–	–	–	–	NA	–	Multi	95	+	LR	N	4	Died
	78/M	White	–	–	–	–	–	NA	+	1	105	+	LR	Y	48	Alive
	78/F	White	–	–	–	–	–	NA	+	1	60	–	LR	N	24	Alive
	57/F	White	–	–	–	–	–	NA	–	1	130	+	LR	N	1	Died
Chan et al [24]	58^a^/13 M, 7F	NA	17/20	0	0	8+/20	13/20	NA	20+/20	1	38	NA	LR	NA	5-y survival: 88%	
Wada et al [11]	62^a^/10 M, 1F	NA	0/11	11/11	/	6/11	NA	NA	NA	NA	22	1+/11	LR	NA	5-y survival: 100%	

Calderaro et al. mentioned 13 cases of LEL-HCC treated with liver resection for hepatocellular carcinoma, but no relevant information was provided.

*F* female, *M* male, + positive, − negative, *AFP* a-fetoprotein, *NA* not available, *HBV* hepatitis B virus, *HCV* hepatitis C virus, *EBV* Epstein-Barr virus, *no*. number, *R* right liver, *L* left liver, *LR* liver resection, *CT* chemotherapy, *OLT* orthotopic liver transplant, *N* NO, *Y* Yes, *FU* follow-up, *mo*. months, ^a^Indicated as the mean value

**Table 4:** Population and tumor characteristics of patients with LEL-HCC and LEL-CC

Classification	Male	Medianage, years (range)	Race		HBV+	HCV+	Cirrhosis	AFP, > 20 ng/mL	BCLC stage,0/A	Single lesion	Median size, mm (range)	Vascular invasion	EBV+	Treatment	
			White	Asian										Resection	Transplant
LEL-HCC (*n* = 67)	34/54	58 (37–81)	13/20	6/20	23/54	18/54	24/53	21/41	37/43	38/43	38(10–130)	7/28	1/41	59/65	6/65
LEL-CC (*n* = 34)	12/34	57 (19–77)	2/27	25/27	13/34	2/34	5/34	0/4	NA	29/34	35(15–120)	NA	26/34	29/31	0/31

Data are given as number/total. *AFP* a-fetoprotein, *HBV* hepatitis B virus, *HCV* hepatitis C virus, *EBV* Epstein-Barr virus, *NA* not available

Macroscopically, the tumor has good boundaries and encapsulation and is gray-white and soft to touch [9]. The median size of the tumor is 38 mm (10–130). Histologically, the cancer cells are poorly differentiated, composed of atypical cells, with prominent nuclei and nucleoli. A large number of lymphocytic infiltrates are characteristic and common in LEL-HCC, which can be used to distinguish it from typical HCC [11, 18]. Most infiltrating lymphocytes are T cells, mainly CD8+ T cells, and local CD20+ B cells are also found [18].

The diagnosis of LEL-HCC mainly depends on pathological methods. There is no specificity in the clinical manifestations and imaging examination of patients. Most patients are confirmed by pathological diagnosis and immunohistochemistry after surgical treatment.

In terms of survival and prognosis, the prognosis of LELC is better than that of typical liver cancer. According to Chan et al [24], LEL-HCC has better overall survival (5-year survival 94.1%: 63.9%; P <0.05) and progression-free survival (5-year survival 87.8%: 46.6%, P<0.05) than HCC. This research suggested that LEL-HCC is an independent prognostic factor for overall survival.

### Lymphoepithelioma-like cholangiocarcinoma(LEL-CC)

LEL-CC is one of the pathological classifications of LELC. To date, 34 cases of LEL-CC have been reported (see Table 5). The available demographics of the patients and features of the tumors are displayed in Table 4. Overall, there are fewer cases than for LEL-HCC. According to the current literature analysis, patients with LEL-CC are mainly Asian women, with 92% of patients being Asian, with a median age of 57 years (46–64). Thirteen patients had HBV infection, accounting for 39%, and 2 patients had HCV infection, accounting for 6%; 5 patients had cirrhosis, accounting for 15%. AFP was normal, and 24 patients had EBV infection, accounting for 76%. Twenty-eight patients received surgical treatment, accounting for 93%.

**Table 5:** Reported cases of LEL-CC

Reference	Age/Sex	Race	HBV	HCV	EBV	Liver cirrhosis	Tumor no.	Tumor size(mm)	Treatment	FU, months	Outcomes
Gearty et al [26]	28/F	Asian	+	–	+	NA	1	40	CT	9	Died
Ding et al [27]	75/F	Asian	–	–	+	–	1	15	LR	3	Alive
Ling et al [28]	64/M	Asian	+	–	+	–	1	20	LR	11	Alive
	40/M	Asian	+	–	+	–	1	35	LR	32	Alive
Zhang et al [29]	38/F	Asian	+	–	+	–	1	28	LR	6	Alive
Shih et al [30]	77/F	White	+	–	–	+	2	17	LR	28	Alive
Tan et al [31]	22/M	White	NA	NA	+	NA	Multi	NA	CT	NA	Died
Adachi et al [32]	64/M	Asian	–	–	–	–	1	52	LR	3	Alive
Chen et al [33]	67/F	Asian	–	–	+	–	1	50	LR	<1	Died
	41/M	Asian	+	–	–	+	1	30	LR	8	Alive
Henderson-Jackson et al [34]	63/F	Asian	–	–	+	–	1	40	LR	6	Alive
Hsu et al [35]	47/F	Asian	–	–	+	–	2	120	LR	48	Died
Huang et al [36]	60/F	Asian	–	–	+	–	1	35	NA	24	Alive
Hur et al [37]	57/F	Asian	–	–	–	–	1	20	LR	60	Alive
Jeng et al [38]	42/M	Asian	–	–	+	–	1	30	LR	84	Alive
	67/F	Asian	–	–	+	–	1	30	LR	7	Alive
	50/M	Asian	–	–	+	–	1	40	LR	16	Alive
	50/F	Asian	–	–	+	–	1	40	LR	2	Alive
Kim et al [39]	64/M	Asian	–	+	–	+	1	20	LR	NA	NA
Labgaa et al [40]	58/M	Asian	+	–	+	–	1	22	LR	61	Alive
Lee [41]	79/M	Asian	+	–	–	+	2	35	LR	54	Alive
Liao et al [42]	35/F	NA	+	–	+	+	1	16	LR	NA	NA
Ortiz et al [43]	19/F	White	–	–	+	–	1	55	LR	44	Died
Szekely [44]	61/M	NA	–	–	–	–	1	60	NA	11	Alive
Vortmeyer et al [45]	71/F	White	–	–	+	–	2	50	NA	36	Alive
Chan et al [46]	53/F	Asian	+	–	+	+	1	16	LR	165	Alive
	40/F	Asian	+	–	+	–	1	75	LR	56	Alive
	57/F	Asian	–	–	+	–	1	71	LR	128	Alive
	56F	Asian	–	–	+	–	1	60	LR	69	Died
	59/F	Asian	+	–	+	–	1	60	LR	72	Alive
	45/F	Asian	–	–	+	–	1	30	LR	71	Alive
	57/F	Asian	–	–	+	–	1	30	LR	58	Alive
Aosasa et al [47]	65/F	NA	–	+	–	–	1	64	LR	20	Alive
Min et al [48]	46/M	Asian	+	–	+	–	1	27	LR	84	Alive

*F* female, *M* male, + positive, − negative, *NA* not available, *HBV* hepatitis B virus, *HCV* hepatitis C virus, *EBV* Epstein-Barr virus, *no*. number, *LR* liver resection, *CT* chemotherapy, *OLT* orthotopic liver transplant, *FU* follow-up, *mo*. months

Macroscopically, the tumor tissue is white-brown, firm and without capsule and similar to typical ICC [10]. The median size of the tumor is 35 mm (15–120). Histologically, there are two different components in LEL-CC; LEL-CC and typical cholangiocarcinoma exist at the same time, or only LEL-CC is present, and the histological differences are in sharp contrast with LEL-HCC [40, 42]. The infiltrating lymphocytes are mainly CD3+ T cells, local CD20+ B cells and CD138+ plasma cells [10, 49].

Similar to LEL-HCC, the diagnosis of LEL-CC mainly depends on pathological methods. The clinical manifestations and imaging examination of patients are not specific. For the vast majority of patients, surgical treatment and later pathological diagnosis are performed.

In terms of prognosis and survival, LEL-CC-related data are limited. A retrospective study [46] compared 7 cases of LEL-CC with 11 cases of stage matched ICC, indicating no significant difference in DFS (5-year survival was 57.1%; 11.7%; *P* = 0.1) and that total survival was significantly higher than that of ICC (5-year survival was 100%; 13.2%; *P* = 0.003).

## Discussion

It is worth noting that two cases of LELC with both hepatocellular carcinoma and cholangiocarcinoma are found in the literature. We call it “combined lymphoepithelioma-like hepatocellular carcinoma and cholangiocarcinoma” (cLEL-HCC-CC).

The first study [50] reported a 62-year-old woman who was admitted to the hospital because of right upper abdominal drop pain with hepatitis B. Laboratory examination showed that AFP was 394.90 ng/ml (0–7 ng/ml). The imaging examination showed that the tumor in the VI segment of the liver was approximately 50 mm. The patient received surgical treatment. According to postoperative pathological and immunohistochemical markers, there were two epithelial cell groups in the tumor, one of which expressed CKs AE1/AE3, hepatocyte, TTF1, AFP, and CD10 and another expressing CK7 and CK19. The former indicates a source of hepatocytes, and the latter indicates a source of the bile duct. This is the first report about cLEL-HCC-CC in English literature.

The second study [51] reported a 40-year-old man who was admitted to the hospital because of the presence of a liver tumor based on physical examination with hepatitis B. Laboratory examination showed that AFP was 4539.2 ng/ml. CT revealed an irregular low-density shadow, of approximately 21 × 21 mm, with uneven internal density and an unclear boundary, in the lower border area of segments V and VI of the liver. After enhancement, the lesions showed irregular enhancement in the arterial phase without obvious lymphadenopathy, and we considered it to be liver cancer. After the operation, the tumor cells were found to be heteromorphic, with a large number of mature lymphocytic infiltrates. Immunohistochemistry showed hepatocyte positivity for CK7, CK19, and CK8/18, and in situ hybridization demonstrated negativity for EBER. Immunohistochemistry suggested origins of hepatocytes and the bile duct. After the operation, the patient was given 2 rounds of hepatic artery intubation chemotherapy, and no tumor recurrence or metastasis was found after close follow-up for 30 months. The patient was in good condition.

The first case we reported was pathologically diagnosed as LELC after surgical treatment. Microscope revealed hepatocellular carcinoma and cholangiocarcinoma. Immunochemistry markers were as follows: CK18 (3+), CK19 (1+), CK7 (1+), GPC3 (3+) and hepatocytes (2+). Combined with the reports of the above two cases, we believe that this case can also be diagnosed as cLEL-HCC-CC.

We compared the clinical data and pathological immunohistochemical factors of three patients with cLEL-HCC-CC. The results are compiled in Table 6. Two of the patients were Asian males; the 3 patients had HBV infection, and AFP was increased. All patients had a single tumor less than or equal to 50 mm. All patients underwent surgery. The results of immunohistochemistry showed that the tumors all originated from hepatocytes and the bile duct; the 3 tumors were positive for hepatocytes, CK7 and CK19. The longest follow-up period was 30 months, and the survival of the patients was good. Compared with mixed liver cancer, cLEL-HCC-CC is very rare. In fact, it is difficult to retrieve relevant information in the literature. In the future, more research and reports are needed to improve knowledge about this special pathological type.

**Table 6:** Clinical and pathological data of cLEL-HCC-CC

References	Sex	Age, years	Region	HBV	HCV	AFP, > 7 ng/mL	No. of lesions	Size, mm	Treatment	FU, mo.	Outcome	Immunohistochemical markers					
												AFP	Hepatocyte	CK7	CK19	CK18	EBER
First case	M	62	Asian	+	–	+	1	28	LR	13	AWOD	–	+	+	+	+	–
Filotico et al [50]	F	62	NA	+	–	+	1	50	LR	NA	NA	+	+	+	+	NA	NA
Wei et al [51]	M	40	Asian	+	–	+	1	21	LR	30	AWOD	–	+	+	+	+	–

*F* female, *M* male, + positive, − negative, *HBV* hepatitis B virus, *HCV* hepatitis C virus, *NA* not available, *No*. number, *AFP* a-fetoprotein, *AWOD* alive without disease, *FU* follow-up, *mo*. months, *LR* liver resection, *EBER* EBV-encoded RNA

Since the attention of LELC, there have been many reports about LELC in recent years. According to our learning, 101 cases of LELC patients have been reported. We reported three patients at one time, reviewed the literature and summarized the LELC. We have studied and reported cLEL-HCC-CC for the first time, which may be a new pathological type and should be concerned. But our article is still insufficient. In fact, there are only two new cases, and the third case we have reported before. Because of the outcome indicators of patients, we have made a brief report.

LELC was acknowledged by the WHO as a distinctive variant of liver cancer in 2010. The diagnosis requires pathological observation of a large amount of lymphocyte infiltration, but the density of lymphocyte infiltration needed for diagnosis has not been determined. In 2019, WHO updated the key histological feature of LELC, which is that lymphocytes outnumber tumor cells in most fields on H&E staining. In addition, WHO also proposed a new subtype of hepatocellular carcinoma, called lymphocyte-rich type [52]. We summarized the differences between LEL-HCC and hepatocellular carcinoma, lymphocyte-rich type. First, they differ in degree of differentiation, with LEL-HCC being poorly differentiated and lymphocyte-rich type being moderately or well differentiated. Secondly, there are differences in immunohistochemical factors. Hepatocyte and arginase (Arg) are usually positive in lymphocyte-rich type, while markers of hepatic differential of LEL-HCC may be negative or focal [53]. Both second case and third case reported by us were diagnosed with LEL-HCC rather than hepatocellular carcinoma, lymphocyte-rich type. The histology of two cases was poorly differentiated and both Hepatocyte and Arg were negative. The pathological features supported our diagnosis.

We also discussed how to distinguish LEL-HCC from LEL-CC, which is similar to the diagnosis of HCC and ICC. AFP, Hepatocyte and GPC3 proteins are specific immunohistochemical factors for the diagnosis of hepatocellular carcinoma, while CK19 and CK7 are specific immunohistochemical factors for the diagnosis of cholangiocellular carcinoma. In most cases, pathological diagnosis depends on these specific immunohistochemical factors, but there are some special cases. For example, both AFP and Hepatocyte in case 2 were negative, CK19 and CK7 were also negative, but CK18 was positive [54]. Combined with the microscopic characteristics of hepatocellular carcinoma with low degree of fibrosis and poor differentiation, we diagnosed it as hepatocellular carcinoma. In case 3, AFP and CK19 was positive. Combined with the microscopic characteristics of hepatocellular carcinoma with low degree of fibrosis and poor differentiation, hepatocellular carcinoma was also diagnosed. The expression of CK19 in hepatocellular carcinoma suggests that the prognosis may be poor [55], which is consistent with the actual situation of the patient, who has already developed lymph node metastasis.

Therefore, the diagnosis of LELC is mainly based on pathological methods. Under the microscope, atypical tumor cells with low differentiation or undifferentiation, characterized by a large number of lymphocytic infiltrates, are observed. LELC can be distinguished from typical liver cancer according to the above pathological characteristics. Additionally, LELC can be divided into two types according to microscopic observation and expression of immunohistochemistry factors: LEL-HCC and LEL-CC. LELC can be diagnosed by pathological methods, but this is limited to patients who have received hepatectomy, liver puncture or liver transplantation.

In molecule research, EBV infection is an important cause of nasopharyngeal carcinoma (NPC) [56]. Whether EBV infection occurs in LELC is currently a concern. According to our statistical data (Table 2), only one patient with LEL-HCC had EBV infection; 25 patients with LEL-CC had EBV infection, accounting for 76%. This indicates that the occurrence and development of LEL-CC may be closely related to EBV infection. This finding is worthy of further study to explore whether EBV is directly involved in the development of LEL-CC or whether it provides a protective factor by causing a special immune response, improving its prognosis. In addition, a number of studies have reported inflammatory cells. In LELC, the infiltrating lymphocytes are composed predominately of CD4+ and CD8+ T cells [53], along with scattered germinal centers that contain B cells [10]. The difference of lymphocyte phenotype between LEL-HCC and LEL-CC needs further study.

The clinical manifestations of LELC patients are not special. Most patients have physical examination findings, and some of them have right upper abdominal pain or chronic cholecystitis symptoms [16, 21, 50]. Nonetheless, because of its nonspecific clinical manifestations, it is difficult to diagnose LELC before surgery.

The prognosis of patients with LELC is better than that of typical liver cancer [52], which may be related to a large amount of lymphocyte infiltration [24]. The second patient reported by us was admitted to the hospital at 1 year after the operation because of abdominal pain and recurrence. The patient received radiotherapy later. At present, the follow-up period is 61 months. The patient has good survival and no metastasis or recurrence. The DFS was 23 months. The third patient, who received liver puncture before the operation to indicate lymph node metastasis, had local recurrence after the operation. The patient received nine cycles of chemotherapy and one cycle of radiotherapy. At the 24-month follow-up. The patient was deceased. These two cases suggest that even locally advanced LELC with postoperative recurrence and preoperative lymph node metastasis should be actively treated and treated, and a longer survival period may ensue. However, a more convincing prospective experimental study is needed to explore the prognosis of LELC.

We summarize the diagnosis and treatment strategy of LELC. First, because LELC is a relatively rare liver cancer variant with a low incidence rate, it is necessary to consider the possibility of LELC in the process of the diagnosis and treatment of liver cancer. Moreover, it is suggested that the treatment strategy should be formulated under MDT. Second, preoperative EBV can be perfected; if it is positive, it will better support the diagnosis of LELC. Third, in the case of advanced liver tumors, it is recommended that liver biopsy be performed and the relevant pathological diagnosis be perfected. If LELC is present, it should be treated actively. Fourth, when LELC is present according to postoperative pathological results, because it may have a better prognosis, even with local recurrence and metastasis, active intervention and treatment is still recommended to achieve the possibility of long-term survival.

## Conclusion

At present, the study of LELC is still in progress, but preliminary analysis shows that it is a distinctive variant of liver cancer characterized by large lymphocyte infiltration. LELC can be divided into LEL-HCC, LEL-CC and cLEL-HCC-CC, with unique epidemiological and pathological characteristics. Its diagnosis mainly depends on pathological methods, and treatment mainly depends on surgery.

To date, 41 studies have been published, from 1998 to 2020, and 67 cases of LEL-HCC and 34 cases of LEL-CC have been reported. According to the literature, LELC has a good prognosis, and even mixed pathological type or locally advanced cases of LELC with local recurrence and distant metastasis may still have long-term survival. Whether EBV affects the development and prognosis of LELC is not yet clear. Prospective studies are needed to explore the prognosis of LELC.

## Data Availability

All data generated or analyzed are included in this published article.

## Abbreviations

LELC: LYMPHOEPITHELIOMA-like carcinoma
HBV: Hepatitis B virus
HCV: Hepatitis C virus
EBV: Epstein-Barr virus
LEL-HCC: Lymphoepithelioma-like hepatocellular carcinoma
LEL-CC: Lymphoepithelioma-like cholangiocarcinoma
cLEL-HCC-CC: Combined lymphoepithelioma-like hepatocellular carcinoma and cholangiocarcinoma
HCC: Hepatocellular carcinoma
ICC: Intrahepatic cholangiocarcinoma
MDT: Multidisciplinary team
MRI: Magnetic resonance imaging
F: Female
M: Male
AFP: a-fetoprotein
NA: Not available
FU: Follow-up
mo: Months
No: Number
DFS: Disease-free survival
LR: Liver resection
CT: Chemotherapy
RT: Radiotherapy
